# Short-Term Heat Stress Differentially Affects the Photosynthetic Thermotolerance of Cotyledons and Early Orthotropic Leaves in *Coffea arabica* L. Seedlings

**DOI:** 10.3390/biology14121659

**Published:** 2025-11-24

**Authors:** Tiago Vilas-Boas, Felipe Della Torre, Zachary Dalton, Maria Bernadete Lovato, José Pires de Lemos-Filho, Elizabeth R. Waters

**Affiliations:** 1 Departamento de Botânica, Instituto de Ciências Biológicas, Universidade Federal de Minas Gerais, Avenida Antônio Carlos 6627, Belo Horizonte 31270901, MG, Brazil; tiago.vilas@hotmail.com (T.V.-B.); joseplemosfilho@gmail.com (J.P.d.L.-F.); 2 Department of Biology, San Diego State University, 5500 Campanile Drive, San Diego, CA 92182, USA; zpd703@gmail.com; 3 Departamento de Genética, Ecologia e Evolução, Instituto de Ciências Biológicas, Universidade Federal de Minas Gerais, Avenida Antônio Carlos 6627, Belo Horizonte 31270901, MG, Brazil; bernadete.lovato@gmail.com

**Keywords:** *Coffea arabica*, cotyledons, orthotropic leaves, gas exchange, acclimation, heat stress, photosynthesis

## Abstract

Climate change is increasing the frequency and intensity of heat waves. Increased temperatures can negatively impact plant growth and development, and thus decrease plant yield. The heat tolerance of coffee, *Coffea arabica*, is of widespread interest due to the importance of this species. Here, we describe studies on the effects of high temperatures and heat acclimation on the photosynthetic activity and thermal resistance of *Coffea arabica* seedlings. We sought to determine if there were differences between cotyledons and early orthotropic leaves in their response to heat stress. We report that acclimation of *Coffea arabica* seedlings to non-lethal temperatures (38 °C) provides tolerance to more intense heat stress (48 °C) for both cotyledons and early true leaves. In addition, we demonstrate that seedling photosynthesis is maintained after intense heat stress when seedlings are first exposed to a lower heat stress. Further, we show that unlike untreated or control seedlings, pre-treated or acclimated seedlings are able to repair heat-damaged photosynthetic apparatuses after intense heat stress. Importantly, we found significant differences in heat tolerance between cotyledons and early leaves (i.e., orthotropic). Our finding underscores the importance of cotyledons in the heat stress response of young coffee seedlings.

## Abstract (plain language summary)

Coffee is one of the most important crops, supporting millions of smallholder farmers. Coffee is also highly sensitive to high temperatures, which are becoming more common due to climate change. Robust growth at the early seedling stages is crucial for successful plant establishment and later plant growth. In order to better understand how young coffee plants cope with heat stress, we examined young seedlings with cotyledons (seed leaves) and their first true leaves. In our studies, we exposed these seedlings to short periods of high temperature stress, with or without prior mild heat treatment. Our results showed that seedlings that experienced mild heat before extreme heat were better able to maintain photosynthesis and recover afterwards. We also found that cotyledons were more tolerant of high temperatures than the first true leaves. This suggests that cotyledons play an important protective role in young plants when facing stressful conditions. These findings highlight the importance of protecting coffee seedlings during the early stages of growth, when they are most vulnerable to climate stress. Understanding how different parts of the plant respond to heat can help guide future farming practices and may support the development of more resilient coffee crops.

## 1. Introduction

Coffee (*Coffea* spp.) is one of the most important crops of the intertropical zone (20–25° N to 24° S) [1,2]. This region has at least 25 million smallholder farmers in over 75 countries who depend on the coffee crop for their livelihoods [3,4]. Suitable areas for *Coffea arabica* cultivation have mean annual temperatures ranging from 18 to 23 °C, with annual precipitation levels between 1500 and 2000 mm [5]. However, ongoing climate change has resulted in significant changes to intertropical climate patterns [6]. This, in turn, has resulted in significant reductions in coffee fitness and productivity [7,8,9]. As we look ahead, it is highly likely that coffee production will be further reduced due to increasing temperature stress [10]. Most climate models agree that a rise of 1.5 °C in mean global temperature is unavoidable. This moderate increase, which is not the worst-case scenario [11], will result in a loss of at least 30% of the areas currently suitable for coffee cultivation [12,13,14,15]. Previous studies have shown that morphophysiological acclimation in coffee seedlings to harsh climatic scenarios, such as elevated CO_2_ concentration, repeated drought cycles, longer exposure to high temperatures, or drought and heat stress combination [16,17,18,19], may mitigate some of the impacts of our changing climate. As seedlings represent the stage at which coffee plants begin their field establishment, understanding their acclimation potential is particularly relevant for coffee farming practices, since the vigour and thermotolerance of young plants directly influence plantation success and long-term productivity [20].

Compared to other abiotic factors, temperature has quite a large impact on coffee yield. Therefore, temperature is an important factor for farmers when choosing where to grow coffee. In particular, high temperatures (above 35 °C) during both fruit filling and leaf expansion [15,21] reduce *C. arabica* yield and decrease beverage quality [3,22]. Coffee, like most plants, is negatively impacted by heat stress primarily because photosynthesis is highly sensitive to high temperatures [23,24]. Heat stress reduces a plant’s photosynthetic activity due to the accumulation of heat-induced reactive molecules that damage the photosynthetic apparatus and other cellular components [25,26]. In addition, high heat can drive changes in lipid membrane fluidity and cause the inactivation of photosynthetic enzymes [27,28]. More detailed reviews of the heat shock response and plant heat stress research can be found in the following references [23,24,29,30,31,32,33].

Plants can attain what is termed acquired thermotolerance after exposure to high (38 °C) but non-lethal temperatures [34,35]. Acquired thermotolerance provides organismal heat tolerance for short or extended periods, and this general phenomenon has been demonstrated in a wide range of plants [29]. Detailed studies in *Arabidopsis thaliana* have demonstrated higher survival rates in acclimated seedlings (i.e., subjected to acclimation at 38 °C before exposure to 45 °C) compared to plants exposed to basal stress (no pretreatment) [36,37]. Similarly, seedlings of *Boechera* spp. (Brassicaceae) from arid environments, that had first undergone acclimation before extreme stress, exhibited reduced visual leaf damage and higher quantum yield of photosystem II (ΦPSII) compared to non-acclimated plants [38,39].

Previous studies in *Coffee arabica* have demonstrated that gradual increases in temperatures up to 37 °C did not result in acclimation or acquired thermotolerance, as measured by the protection of photosynthetic activity [16]. Several studies have demonstrated the sensitivity of photosynthetic activity in coffee to high temperatures. Researchers have found that coffee plants exposed to extreme stress, 42/34 °C (day/night), showed photoinhibition and a reduced net photosynthesis rate (*P*_n_) [16,40]. In another study, researchers exposed *C. arabica* plants to extreme heat (above 45 °C) without acclimation and found decreased *P*_n_ and the maximum quantum yield (Fv/Fm) compared to non-stressed plants [41,42]. In addition, it was found that at least 15 days of recovery at non-stress temperatures was required to recover the net photosynthesis rate and Fv/Fm levels of non-stressed plants [41]. Taken together, these previous studies suggest that heat stress will result in substantial reductions in carbon acquisition and plant growth, and they suggest that early heat stress of seedlings could have large and long-lasting impacts on coffee production.

The heat tolerance of photosystem II (PSII) in plants can be assessed through assays that expose leaf discs to increasing temperatures, monitoring PSII stability by chlorophyll fluorescence parameter Fv/Fm until it collapses [27,43]. In *C. arabica* plants grown under greenhouse and supplementary light [41,44], 49 °C was the temperature at which the Fv/Fm of leaf discs decreased by 50% compared to the initial value (T_50_). However, Ref. [45], evaluating *C. arabica* varieties grown under high or low sunlight, found a T_50_ above 54 °C and a T_15_ (indicating a 15% decrease in Fv/Fm compared to initial values) close to 49 °C, also using leaf discs. These results suggest that photosystem II heat tolerance (PHT) depends on the acclimation conditions. Additionally, *C. arabica* PHT varies according to the seasons and leaf ontogeny, with young leaves presenting lower PHT than mature ones [44,46]. These data pointed to the high plasticity of PHT in coffee plants, and several important questions remain unanswered. Importantly, the effects of extreme heat on PSII chlorophyll fluorescence and critical boundaries T_15_ and T_50_ plasticity remain unclear.

Even though the early stages of plant development are essential for successful plant establishment, heat stress effects on the photosynthesis of early seedlings remain poorly understood. Previous studies on the impact of high temperatures on wheat and cotton cotyledons showed lower heat injury in acclimated plants [47]. It has been proposed that cotyledons could have a key role in the stress tolerance of *Quercus variabilis* seedlings under drought stress by Zhao et al. [48]. Given the fundamental role of the cotyledons and the early-emitted leaves in plant development, it is important to know the effects of heat stress on the photosynthesis of these structures.

The cotyledons (cotyledonary leaves) of *C. arabica* emerge 30–45 days after full seed imbibition and are the first photosynthetic tissue that becomes fully independent from the seed endosperm [49,50]. The expansion of the orthotropic leaves begins approximately two months after seed imbibition (personal observation). At this time (two months past germination), coffee seedlings possess cotyledons and at least 4–6 pairs of orthotropic leaves [51]. Knowledge of the photosynthetic performance of coffee at very early development stages under heat stress remains unstudied, as most studies are conducted on coffee plants older than 6 months. Given the importance of cotyledons and early orthotropic leaves in early seedling development and plant establishment and the limited understanding of their photosynthetic performance under heat stress, we aimed to determine how short-term acclimation influences the heat tolerance and photosynthetic recovery of cotyledons and early orthotropic leaves in *C. arabica* seedlings.

We hypothesized that (1) heat acclimation will enhance the heat tolerance (as measured by photosynthetic rate (*P*_n_) and maximum quantum efficiency of PSII: Fv/Fm) and that the cotyledons and early true leaves will respond differently to acclimation; (2) the recovery of photosynthetic performance after heat stress will be higher in acclimated leaves of both types compared to the leaves of non-acclimated seedlings; (3) critical temperature thresholds for PSII impairment (T_15_ and T_50_) will be higher in both types of leaves of acclimated seedlings than in non-acclimated ones, but cotyledons will exhibit greater thermotolerance compared to early orthotropic leaves.

## 2. Materials and Methods

### 2.1. Plant Growth

**Figure 1:**
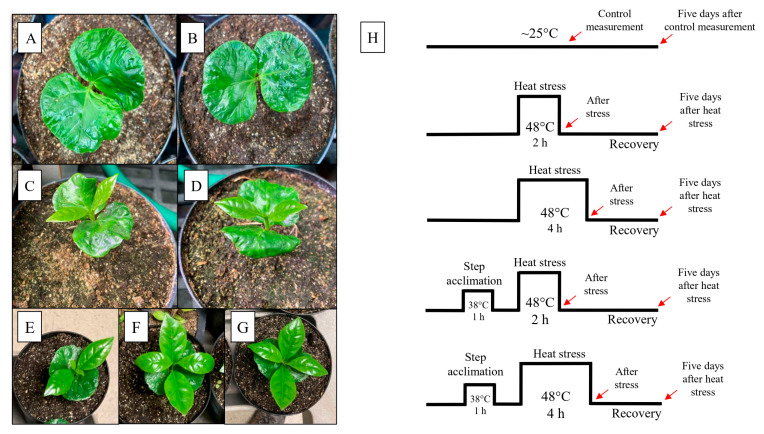
*Coffea arabica* cv. Catuai Amarelo seedlings developmental stages: seedlings with cotyledons ~45 days after seed imbibition (**A**,**B**); first orthotropic leaves pair emergence ~60 days after seed imbibition (**C**,**D**); seedlings with two pairs of first orthotropic leaves between them at the of heat stress 105 days after seed imbibition (**E**–**G**). Representation of control, the non-acclimation (**upper**), and acclimation of plants at 38 °C (**bottom**); previously, the heat stress was at 48 °C for 2 or 4 h (**H**) treatments. Seedlings were placed for 1 h in the growth chamber at 25 °C and 100 μmol m^−2^ s^−1^ of PPFD between acclimation and heat stress. Red arrows indicate the moment of evaluations after heat stress (AS) and five days after heat stress (REC) treatments. Recovery occurred at greenhouse conditions. Control plants were kept under greenhouse conditions during heat stress treatments.

Seeds from *Coffea arabica*, var. Catuaí Amarelo, were obtained from a farmer in Pirajú city, São Paulo, Brazil. Seeds were processed, and those without damage were selected and sterilized with sodium hypochlorite (5%) for 5 min and washed 3 times in deionized water. Seeds were placed in a germination box covered with filter paper, moistened with deionized water as needed, and placed to germinate in a Percival E36L growth chamber (Percival Scientific, Perry, IA, USA) set at 25 °C and 100 μmol m^−2^ s^−1^ photosynthetic photon flux density (PPFD). Following germination, the seedlings were transferred to the greenhouse at San Diego State University (San Diego, CA, USA) with night/day temperatures of 19–28 °C, with humidity between 40 and 50% and supplemental light of ~500 μmol m^−2^ s^−1^ PPFD, at a photoperiod of 14 h/10 h (light/dark). The seedlings were initially transplanted into small pots (0.05 L) for 30 days and then moved to larger pots (3 L) containing a soil (#1, Sungro, Canada): sand mix (1:1; *v*:*v*) for an additional 75 days of growth. The seedlings were randomly reorganized weekly under the light source of ~500 μmol m^−2^ s^−1^ PPFD to avoid any impact from variations in light intensity on plant growth. Regular watering was administered every two days, maintaining the soil near field capacity. Additionally, every 15 days, each pot received supplementation of 50 mL with a half-strength commercial nutrition solution (Jack’s, JR Peters INC., Allentown, PA, USA). At the end of the growth period, totaling 105 days after seed germination, we selected seedlings with cotyledons (CL) and early-emitted orthotropic leaves (EOL) to conduct heat stress experiments (Figure 1A–G).

### 2.2. Heat Stress Treatments

The experimental design, including the temperatures used to assess thermotolerance, is based on the temperature used to evaluate PSII tolerance in coffee [41,44] and in a wide range of other species [27,43]. The choices of experimental protocol and temperatures used permit our findings to be compared to the state of the literature. We conducted a short-term heat stress on coffee seedlings to assess the photosynthetic response of cotyledons (CL) and early-emitted orthotropic leaves (EOL) using standard protocols [29,38,39]. The temperatures used for these heat stress experiments were chosen based on the previous studies described above and the recent finding that a single short-term heat stress of no longer than 2 h at 49 ± 1 °C can severely impair the photosystem II (PSII) of coffee leaf discs [45] and the gas exchange of coffee leaves [41,44].

When plants were 105 days old, they were exposed to heat stress as described below. Coffee seedlings were standardized and divided equally into five groups (*n* = 4): **(1)** group one was exposed to a basal stress for two hours to a temperature of 48 ± 1 °C, [48 °C (2 h)]; **(2)** group two was exposed to a basal stress for four hours at 48 ± 1 °C, [48 °C (4 h)] (Figure 1); **(3)** group three had an acclimation heat stress of 38 °C for one hour, was allowed to recover for 1 h at 25 °C, and then received a heat stress at 48 ± 1 °C for two hours, [38 + 48 °C (1 h + 2 h)]; **(4)** group four also received an acclimation stress of 38 °C for one hour and was then exposed to four hours of heat stress at 48 °C, [38 + 48 °C (1 h + 4 h)] (Figure 1); and **(5)** the control plants were kept at the standard growth temperature of the greenhouse (~25 °C) and did not receive any heat stress. The heat stress treatments were performed in a Percival E36L growth chamber (Percival Scientific, Perry, IA, USA) with a light intensity of 150–200 μmol m^−2^ s^−1^. The relative humidity during the heat stress was 50 ± 5%, and the pots were covered with aluminum foil during heat stress to avoid superheating the roots [41].

### 2.3. Gas Exchange and Chlorophyll Fluorescence Measurement

The chlorophyll fluorescence and gas exchange traits were evaluated on the same day of the heat stress (AS). These seedlings were kept at greenhouse conditions for five days after heat stress to assess the recovery (REC) of the photosynthetic traits evaluated. In both measurement times, AS and REC, the photosystem II heat tolerance (PHT) assay was made after analyzing chlorophyll fluorescence and gas exchange. We conducted the gas exchange analyses with a portable infrared gas analyzer (IRGA) Model LI 6800 (Li-Cor Inc., Lincoln, NE, USA) with an attached chamber (6800-01A) with an aperture of 2 cm^2^. Measurements were taken [23,24] after heat stress, maintaining a block temperature of 26 ± 0.5 °C, relative humidity between 60 and 65%, and a CO_2_ concentration of 400 µmol (CO_2_) mol (air)^–1^. Light intensity was set to 500 µmol m^−2^ s^−1^ inside the chamber using an LED light source with a spectral light composition of 90% red and 10% blue. We assessed the net photosynthetic rate (*P*_n_), transpiration (*E*), stomatal conductance (*g*_s_), and intercellular carbon (C_i_) for both CL (cotyledons) and EOL (early-emitted orthotropic leaves). Three measurements were taken for the same CL and EOL from each coffee seedling. The mean value of each plant was used in the statistical analyses. Water use efficiency (WUE) was determined by the following: WUE = *P*_n_/*E*. Maximum quantum yield (Fv/Fm) measurements were taken after 30 min of dark adaptation, using the Maxi version of the Imaging-PAM (Heinz Walz GmbH, Effeltrich, Germany) with a light-saturating pulse of ~8000 μmol photons m^−2^ s^−1^. Two measurements were taken of CL and EOL from each coffee seedling, and the mean value of each plant was used in the statistical analyses. It is important to note that different CL and EOL from each seedling were utilized for AS and REC. Since we also evaluated the Fv/Fm again in the PHT assay, we verified the percentage decrease in Fv/Fm from heat stress treatments compared to the control.

### 2.4. Photosystem II Heat Tolerance (PHT) Thresholds

In addition to gas exchange and chlorophyll fluorescence analysis, we assessed the photosystem II heat tolerance (PHT) from leaf discs of CL and EOL after heat stress (AS) and after recovery (REC) for all treatments using the assay optimized for coffee leaves described by Vilas-Boas et al. [45]. In this method, three leaf discs (2 cm^2^) were taken from each coffee seedling’s CL and EOL. Total leaf disc was 12 for each type of leaf, with 24 measurements for each treatment. Leaf discs were placed on moistened filter paper inside a polyethylene bag with an attached thermometer. The initial maximum quantum yield (Fv/Fm) was assessed at a laboratory temperature of 25 °C after 30 min of dark adaptation with the JUNIOR-PAM fluorometer (Heinz Walz GmbH, Effeltrich, Germany). Subsequently, we submerged the set of leaf discs in a water bath (Isotemp 210, Fisher Scientific, Marietta, OH, USA) to the temperature rising, and measurements were taken at 35 °C, 38 °C, 41 °C, 44 °C, 47 °C, 50 °C, 53 °C, 56 °C, and 59 °C. The same leaf disc was evaluated at each temperature. To do this, the leaf discs were acclimated for 3 min at each temperature before the Fv/Fm measurements were taken. Through the ramping assay, we can identify the temperatures that led to a 15% (T_15_) and 50% (T_50_) reduction in the initial Fv/Fm at the laboratory temperature of 25 °C. The critical temperature threshold, T_15_, denotes the inflection point between the slow and fast phase of the decrease in 15% in Fv/Fm with the temperature increase, and T_50_ represents the temperature causing a 50% reduction in Fv/Fm compared to the initial Fv/Fm values [43].

### 2.5. Data Analysis

With the data obtained from the PHT assay, T_15_ and T_50_ were determined utilizing the “car” package and the logistic nonlinear least squares model with the “nls” function in R version 2022.12.0 [43,52]. A T_15_ value is equal to a 15% decrease in Fv/Fm compared to initial values. T_50_ value is equal to a 50% reduction in Fv/Fm compared to initial values. For 1) [48 °C (2 h)], 2) [48 °C (4 h)], 3) [38 + 48 °C (1 h + 2 h)], and 4) [38 + 48 °C (1 h + 4 h)], heat treatments T_15_ and T_50_ were calculated by fitting a logistic nonlinear model using the average Fv/Fm of the control group as the standardized starting point of each response curve. We used the average T_15_ and T_50_ values for each plant in the statistical analysis since each coffee seedling was considered our experimental unit (*n* = 4).

Unpaired T-test (*p* < 0.05) was performed to compare T_15_ results AS. The response variables *P*_n_, *E*, *g*_s_, C_i_, WUE, Fv/Fm, T_15_, and T_50_ were submitted to the Shapiro–Wilk normality test (*p* < 0.05) and Barlett’s test for homoscedasticity (*p* < 0.05). We analyzed the data collected after heat stress (AS) and five days of recovery after stress (REC) separately by one-way ANOVA, which compared the following treatments: Control, [48 °C (2 h)], [48 °C (4 h)], [38 + 48 °C (1 h + 2 h)], and [38 + 48 °C (1 h + 4 h)]. We used post hoc Tukey tests (*p* < 0.05) for pairwise comparisons, and ANOVA analysis when four treatments were analyzed. To contrast CL and EOL response to heat stress, a paired T-test (*p* < 0.05) was used to compare the response variables: *P*_n_, *E*, *g*_s_, C_i_, WUE, Fv/Fm, T_15_, and T_50_. Again, measurements at AS and REC from CL and EOL, or leaf discs in PHT assay, were analyzed separately by paired T-test. For those, the T-test analysis treatment replications were four (*n* = 4). All tests were performed, and the Figures were made using GraphPad Prism version 8.0.2 (San Diego, CA, USA).

## 3. Results

### 3.1. Analysis of Gas Exchange and PSII Responses to Heat Stress and Recovery Reveal Differential Acclimation Between Leaf Types

**Figure 2:**
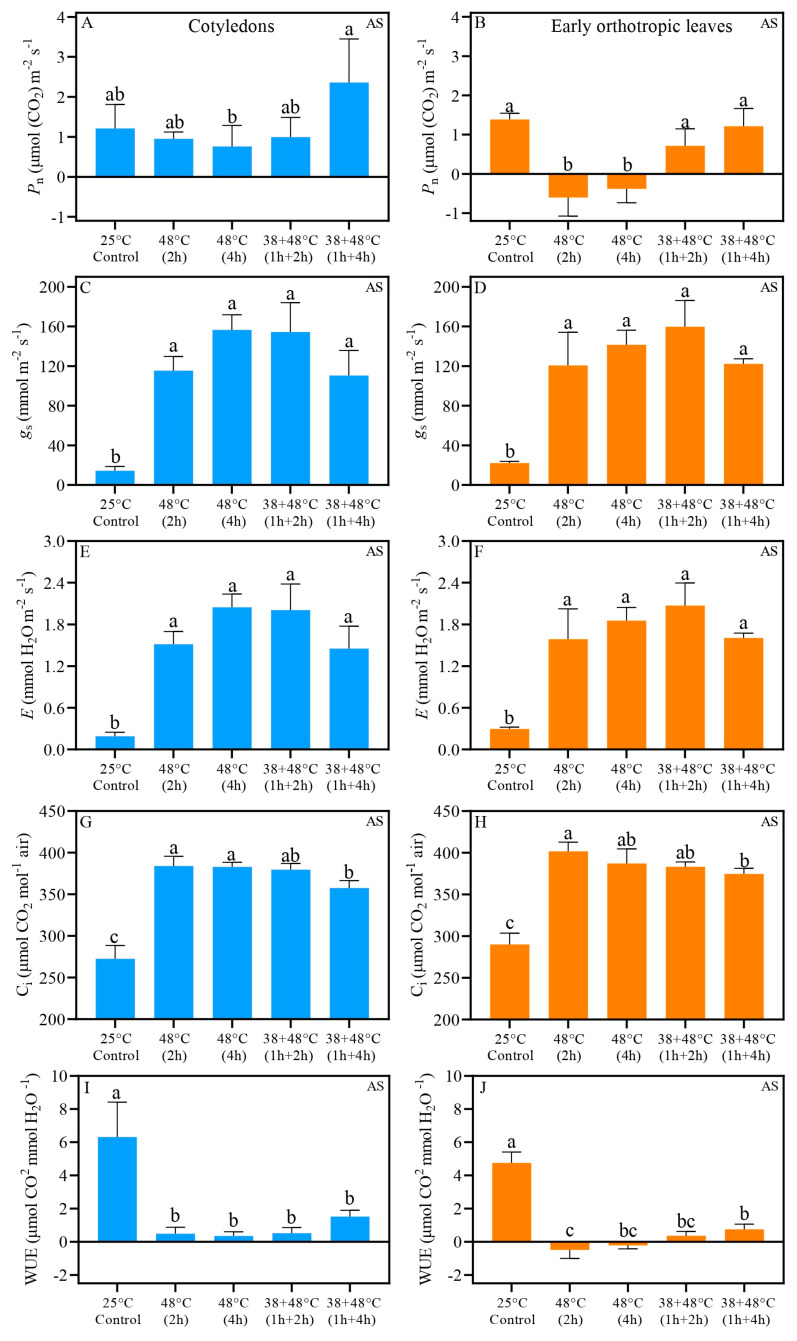
Gas exchange results of *Coffea arabica* cotyledons (blue bars) and orthotropic leaves (orange bars) after heat stress (AS). *P_n_*—net photosynthetic rate (**A**,**B**); *g_s_*—stomatal conductance (**C**,**D**); *E*—transpiration (**E**,**F**); C_i_—Intercellular carbon (**G**,**H**). WUE—water use efficiency (**I**,**J**). Different lowercase letters represent that the means are statistically different by one-way ANOVA and Tukey test (*p* < 0.05). Supplementary Table S1 shows a summary of one-way ANOVA results. *n* = 4; error bars = Standard deviation (SD).

**Figure 3:**
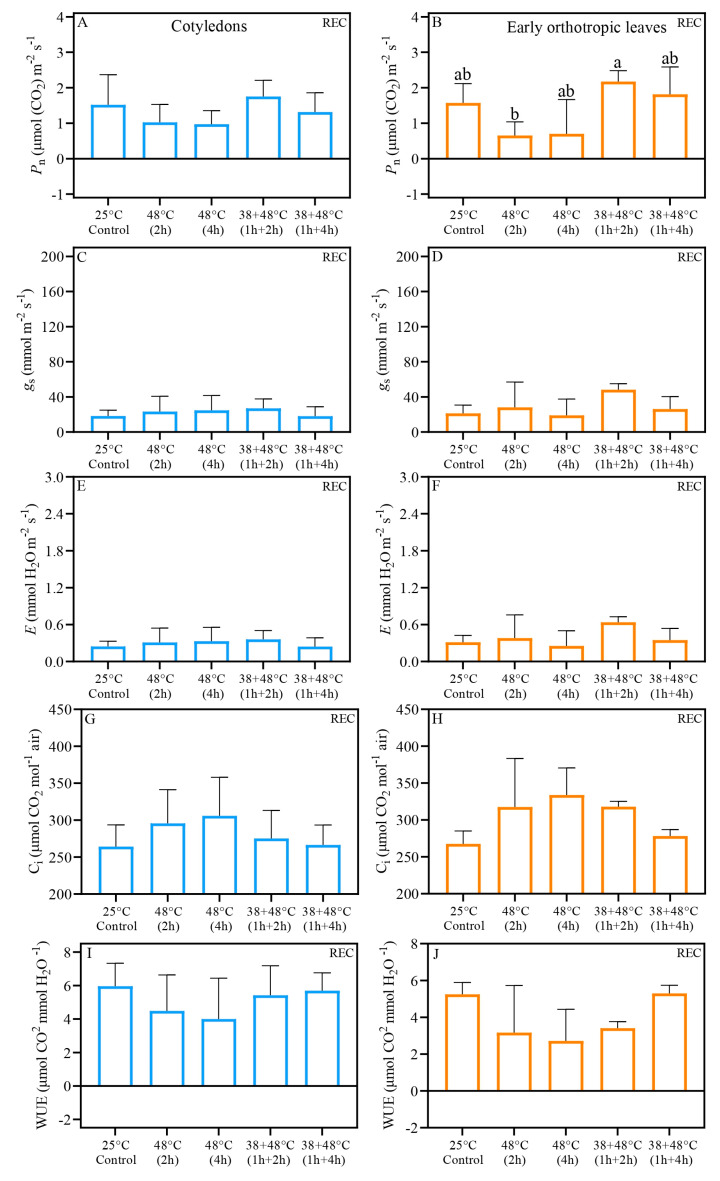
Gas exchange results of *Coffea arabica* cotyledonary leaves (blue bars) and orthotropic leaves (right side—orange bars), five days of recovery after heat stress (REC). *P_n_*—net photosynthetic rate (**A**,**B**); *g_s_*—stomatal conductance (**C**,**D**); *E*—transpiration (**E**,**F**); C_i_—intercellular carbon (**G**,**H**). WUE—water use efficiency (**I**,**J**). Different lowercase letters represent that the means are statistically different by one-way ANOVA and Tukey test (*p* < 0.05). Supplementary Table S2 shows a summary of one-way ANOVA results. *n* = 4; error bars = Standard deviation (SD).

**Figure 4:**
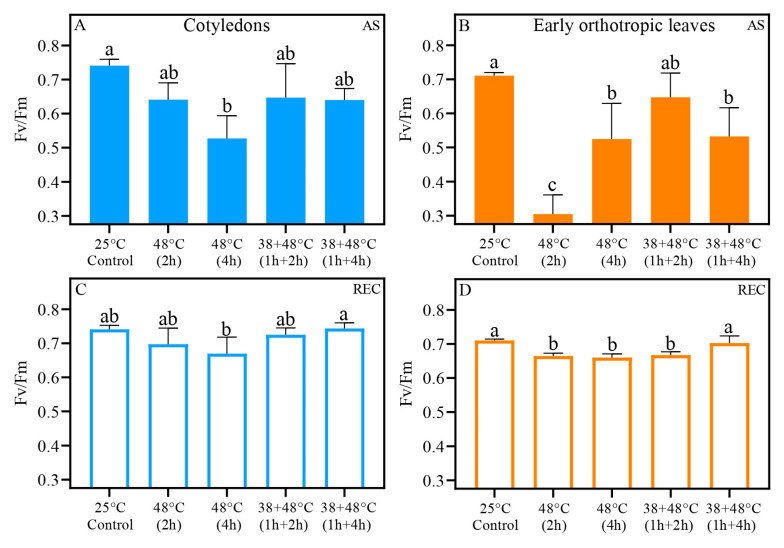
The maximum quantum yield (Fv/Fm) for *Coffea arabica* cotyledonary leaves (blue bars) and orthotropic leaves (orange bars). Filled bars represent after heat stress (AS) (**A**,**B**), and open bars represent five days after heat stress (REC) (**C**,**D**). Different lowercase letters represent different means by one-way ANOVA and Tukey test (*p* < 0.05); Supplementary Tables S1 and S2 show a summary of one-way ANOVA results. *n* = 8; error bars = Standard deviation (SD).

Analysis of gas exchange and quantum yield of PSII indicates that CL and EOL benefit from acclimation prior to heat stress. Further, we demonstrate that the CL and EOL do not always have identical responses to heat stress. The *P*_n_ (net photosynthetic rate) of CL measured immediately after stress (AS) in all heat stress treatments was similar to the control plant, i.e., above 0.70 μmol. When responses between the different types of stresses are compared, we found that the *P*_n_ of CL after [38 + 48 °C (1 h + 4 h)] treatment and the [48 °C (4 h)] differed: *P*_n_, of 2.36 μmol (CO_2_) m^−2^ s^−1^ and 0.76 μmol (CO_2_) m^−2^ s^−1^, respectively (Table 1, Figure 2A). The *P*_n_ values of EOL exposed to heat stress without acclimation, i.e., 48 °C for 2 or 4 h, were negative: *P*_n_ values of −0.60 and −0.38 μmol (CO_2_) m^−2^ s^−1^, respectively. In contrast, when plants were acclimated to heat stress, the EOL maintained control-like *P*_n_ levels (Table 1, Figure 2B). Both the CL and EOL showed large, at least a 6-fold increase in stomatal conductance (*g*_s_) and transpiration (*E*), compared to the control, with *g*_s_ ranging 110 and 122 mmol mm^−2^ s^−1^, and *E* ranging 1.45 and 1.55 mmol H_2_O mm^−2^ s^−1^, respectively, across all heat treatments (Figure 2C–F). Control intercellular carbon (C_i_) ranged from 250 to 300 μmol CO_2_ mol^−1^ air in both leaf types, while heat-stressed leaves showed C_i_ levels above 350 μmol CO_2_ mol^−1^ air (Figure 2G,H). Despite the increased C_i_ in all heat-stressed leaves, the acclimation treatment, [38 + 48 °C (1 h + 4 h)], differed significantly from the non-acclimated treatment, [48 °C (2 h)], in both leaf types when measurements were taken immediately after stress (AS) (Figure 2G,H). Water use efficiency (WUE) also decreased substantially in all heat stress treatments compared to the control for both leaf types (Figure 2I,J). However, after the non-acclimated treatment of [48 °C (2 h)] the EOL showed a distinct WUE compared to the acclimated treatment of [38 + 48 °C (1 h + 4 h)]; the WUE values were −0.48 μmol CO_2_ mmol H_2_O^−1^ and 0.76 μmol CO_2_ mmol H_2_O^−1^, respectively (Figure 2I,J). Five days after heat stress (REC) under greenhouse conditions, *P*_n_ values were found to be similar to the control level across all leaf types (Figure 3A,B). Notably, the EOL REC *P*_n_ differed between the non-acclimated treatment of [48 °C (2 h)] and acclimated treatment of [38 + 48 °C (1 h + 2 h)], with values of 0.65 and 2.17 μmol (CO_2_) m^−2^ s^−1^, respectively (Figure 3B). Other REC measurements, i.e., *g*_s_, *E*, C_i_, and WUE, in both CL and EOL were similar to levels found in control plants (Figure 3C–J). Fv/Fm measured with JUNIOR-PAM (Figure S1) show the same pattern than measurements taken with Imaging-PAM (Figure 4).

Control CL and EOL showed higher Fv/Fm values of 0.71 to 0.74 (Figure 4A,B) compared to HS leaves. A large decrease in Fv/Fm of CL was seen in the [48 °C (4 h)] treatment, with a value of 0.52 for Fv/Fm. Other heat stress treatments had Fv/Fm values of approximately 0.64 (Figure 4A). After a [48 °C (2 h)] heat stress, EOL had a low Fv/Fm measurement of 0.30. Not unexpectedly, this value was low compared to the control. But it is was also much lower than the Fv/Fm values for the other HE treatments. In Figure 4B, we show that after (AS), the 48 °C (4 h) and [38 + 48 °C (1 h + 4 h)], both treatments had Fv/Fm measurements of 0.52. The CL recovered Fv/Fm to control levels of 0.74 in REC, except after the [48 °C (4 h)] treatment, where Fv/Fm remained lower at 0.67 (Figure 4C). The EOL showed an increase in Fv/Fm, but only the [38 + 48 °C (1 h + 4 h)] treatment reached control levels (~0.70), while other treatments averaged around 0.66 in REC, all of them around 5% lower than the control (Figure 4D).

### 3.2. Photosynthetic Heat Tolerance Thresholds (T_15_ and T_50_) Vary Between Acclimated and Non-Acclimated Leaves and Between Leaf Types

**Figure 5:**
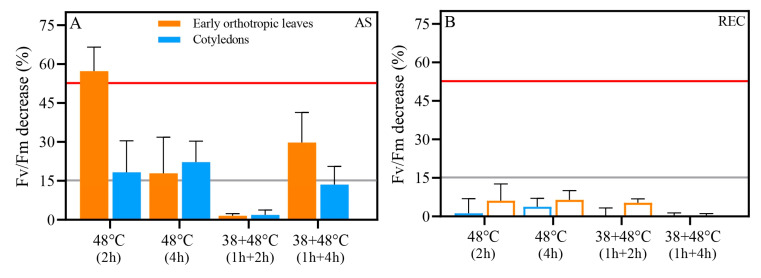
Percentage decrease in the maximum quantum yield (Fv/Fm) in early orthotropic (orange) and cotyledonary (blue) leaves of seedlings subjected to different heat stress regimes after stress (**A**) and after a recovery period (**B**). Bars represent mean ± standard deviation (SD). The grey line indicates a 15% decrease (T_15_ threshold), and the red line indicates a 50% decrease (T_50_ threshold).

Before the PHT assay, we evaluated the percentage reduction in Fv/Fm compared to control levels both after stress (AS) and after recovery (REC). Control values of Fv/Fm were 0.74 and 0.71 CL and EOL (Figure 4). After heat stress treatments, a 15% decrease in Fv/Fm was already recorded for all heat stress groups after stress, except one treatment: [38 + 48 °C (2 h)], where the Fv/Fm decline was below 2% (Figure 5A). The Fv/FM of the EOL from the non-acclimated 48 °C (2 h) treatment declined more than 50% (Figure 5A). As a result, this is the only group with no defined T_15_ or T_50_ thresholds, due to the high damage to the photosynthetic apparatus caused by this treatment. After recovery (REC), all treatments showed Fv/Fm recovery, and differences were below 10% in all heat-stressed groups (Figure 5B).

**Figure 6:**
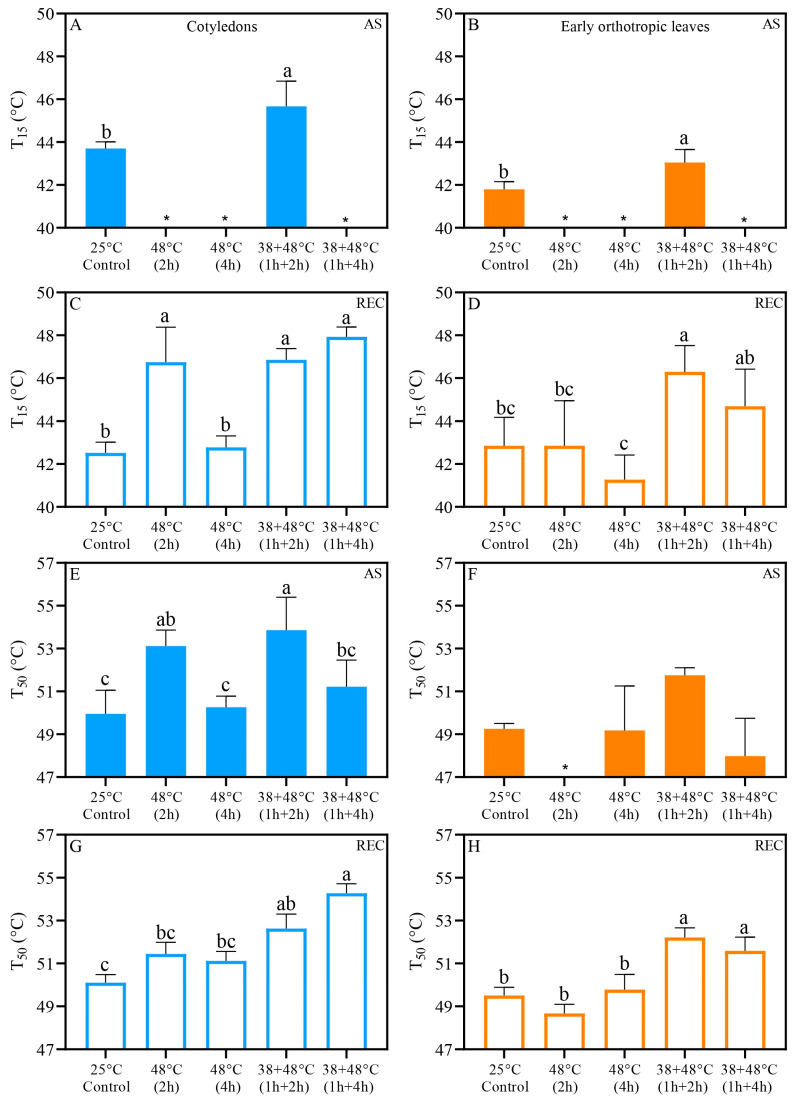
Photosystem II heat tolerance (PHT) parameters T_15_ (**A**–**D**) and T_50_ (**E**–**H**) by leaf discs from *Coffea arabica* cotyledons (blue bars) and orthotropic leaves (orange bars) after heat stress (AS) (filled bars) and five days after heat stress (REC) (opened bars). Different lowercase letters represent different means by one-way ANOVA and Tukey test (*p* < 0.05). Supplementary Tables S1 and S2 show a summary of one-way ANOVA results. *n* = 4; error bars = Standard deviation (SD). Asterisks (*) indicate that T15 or T50 thresholds were already reached by heat stress treatment.

Acclimation to the 38 °C pretreatment [38 + 48 °C (1 h + 2 h)] raised the T_15_ threshold right after stress (AS) in both leaf types, reaching 45.6 °C in CL and 43.1 °C in EOL, relative to control values of 43.7 °C and 41.8 °C, respectively (Figure 6A,B). After the recovery period (REC), only the T_15_ of the CL from the [48 °C (4 h)] treatments were similar to the control, at 42.7 °C and 42.5 °C, respectively. In contrast, the T_15_ values for other heat stress treatments ranged from 46 to 48 °C (Figure 6C). The EOL T_15_ values of acclimated seedlings ranged between 44 and 47 °C, while control and non-acclimated seedlings were lower, with T_15_ ranging from 41 to 43 °C (Figure 6D).

The T_50_ values were calculated and we found that acclimation or exposure to the 38 °C pretreatment resulted in an increase in CL T_50_ to 53–54 °C when leaves were exposed to [48 °C (2 h)] and [38 + 48 °C (1 h + 2 h)] compared to T_50_ around 50–52 °C for leaves exposed to the [48 °C (4 h)], and [38 + 48 °C (1 h + 4 h)] treatments and control conditions when measured immediately after stress (AS) (Figure 6E). The T_50_ for EOL ranged between 48 and 52 °C across all heat stress treatments immediately after stress (AS) and was statistically similar among all groups (Figure 6F). The CL from acclimated seedlings’ heat stress treatments showed higher T_50,_ above 52 °C in REC, with other groups showing T_50_ below 52 °C (Figure 6G). The EOL for the [48 °C (2 h)] and [48 °C (4 h)] treatments exhibited T_50_ similar to the control, around 48–50 °C when taken after recovery REC (Figure 6H). In contrast, all acclimated EOL maintained T_50_ higher than the control at ~52 °C (Figure 6H).

### 3.3. Evaluation of the Photosynthetic Response of Cotyledons and Early-Emitted Orthotropic Leaves Reveals Higher PSII Heat Tolerance in Cotyledons

Statistical analysis (pairwise T-tests) revealed that CL in treatments [48 °C (2 h)], [48 °C (4 h)], and [38 + 48 °C (1 h + 4 h)] showed higher *P*_n_ and WUE compared to the EOL (Table 1). In addition, when AS C_i_ was examined, it was clear that C_i_ was also elevated in CL from [48 °C (4 h)] and [38 + 48 °C (1 h + 4 h)] treatments compared to the EOL (Table 1). Higher *g*_s_ and *E* values were observed in EOL compared to CL, only in the control plants. In addition, the *E* of the EOL for the control plants AS was also higher. There were very few differences between the leaf types in the REC gas exchange variables, with the exception of higher gs and *E* of EOL for treatment [38 + 48 °C (1 h + 2 h)] compared to the CL (Table 1).

We found significant differences between the responses of the two leaf types to heat stress when PSII activity was examined. The Fv/Fm of CL was higher AS after the [48 °C (2 h)] and [38 + 48 °C (1 h + 4 h)] treatments than the Fv/Fm of the EOL after these heat treatments. The Fv/Fm of the [38 + 48 °C (1 h + 2 h)] and [38 + 48 °C (1 h + 4 h)] treatments after recovery (REC) were higher in the CL than in the EOL (Table 2). This pattern is also seen when PSII heat tolerance is examined. The CL have higher T_15_ in control and higher T_50_ of the [38 + 48 °C (1 h + 2 h)] and [38 + 48 °C (1 h + 4 h)] compared to EOL. There was also a significant difference between CL and EOL at REC for T_50_ from the treatment [48 °C (4 h)], with the T_50_ being higher in the CL (Table 2).

## 4. Discussion

The ability of coffee *Coffea arabica* L. plants to withstand higher temperatures and remain productive is of great economic and scientific interest. Global climate change is increasing the frequency and intensity of heat waves, and heat is known to disrupt photosynthesis and to decrease both crop yield and quality. Here, we examine the impact of basal (heat stress with no acclimation) and acclimated (or acquired) heat stress on coffee seedlings. Specifically, we sought to determine if the cotyledons and the early orthotropic leaves were able to tolerate heat stress and if there were differences in their responses to heat with and without acclimation. We have demonstrated that acclimation does assist in coffee seedling heat stress response, but that the two types of leaves found on early seedlings differ in important ways in their tolerance to heat stress.

While the acclimated cotyledons (CL) and early-emitted orthotropic leaves (EOL) of coffee, *Coffea arabica* L, both demonstrated higher net photosynthetic rate (*P*_n_) and chlorophyll fluorescence (Fv/Fm) after heat stress, supporting the hypothesis that acclimation helps maintain photosynthetic function, there were significant differences between these two types of leaves. We found that acclimation led to a slightly faster and more effective recovery in CL and EOL than non-acclimation, indicating that short-term heat exposure can assist in the protection of the photosystems from heat damage and can support photosynthetic recovery after extreme heat stress. Furthermore, when acclimated via a pretreatment of 38 °C, both leaf types displayed increased and longer-lasting photosystem II heat tolerance (PHT), 2–3 °C above non-acclimated plants, further emphasizing the role of acclimation in enhancing thermal resilience. For both types of leaves, two and four hours of high heat (48 °C) without acclimation is highly damaging to photosynthesis. It is highly significant that we found that CL exhibited a higher tolerance in *P*_n_, Fv/Fm, and PHT after heat stress compared to EOL, regardless of type or level of heat stress applied, suggesting inherent structural or functional differences between leaf types that make cotyledon leaves more resilient to heat stress. These findings highlight the value of acclimation for coffee seedling thermotolerance and underscore the differential heat tolerance of leaf types at this early developmental stage.

Acquired thermotolerance generated by short-term exposure to high temperatures has been demonstrated to generate increased plant yield, due to the upregulation of protective genes and photosynthetic traits in crop species (reviewed by Song et al. [37]), the model plant *Arabidopsis thaliana* [36], in wild species [39], and here in the present study for *Coffea arabica* leaves. In contrast to our findings regarding short-term acclimation (one hour at 38 °C) and short-term heat stress exposure (two or four hours at 48 °C) for coffee seedlings, Rodrigues et al. [40] and Martins et al. [16] reported no positive effects of longer acclimation periods on photosynthesis in *C. arabica*. Their findings indicated that exposure of seven days at 37/30 °C (day/night), followed by seven days of exposure to 42/34 °C of leaves from plagiotropic branches, did not result in enhanced protection of the photosynthetic apparatus in *C. arabica* plants. Instead, photoinhibition and lower *P*_n_ were observed [16,40]. A significant difference between our study presented here and these studies is that here we used an acclimation temperature of 38 °C, which is known to turn on the heat-stress response factors [23,36]. It is likely that in studies by Rodrigues [16,40] and Martins [16,40], the acclimation temperatures were not high enough to stimulate the heat shock response. It has been shown that while most plants benefit from acclimation at non-lethal temperatures (38 °C), and that this type of acclimation can increase heat tolerance, there is a limit to the ability of acclimation at 38 °C to prevent photosynthetic damage and cell death [23,39]. The contrast between our results and the other coffee studies cited above suggests that the temperature of the acclimation and the duration of the intense heat stress play crucial roles in determining differences in the photosynthetic response of *C. arabica* in seedlings and adult plants.

Here, we report important similarities and differences in parameters related to water use efficiency (WUE), stomatal conductance (*g*_s_), and E (transpiration rate) between the acclimated and non-acclimated seedlings. The responses of *g*_s_, *E*, and WUE showed similarities between acclimated and non-acclimated seedlings, with all these parameters returning to the control level at REC. Increases in *g*_s_ and *E*, typical responses of well-hydrated plants under heat stress, are associated with maintaining leaf temperature and supporting a positive *P*_n_ under these conditions [53,54]. The WUE, a trait used to measure plant biomass gain relative to the water used by a plant [55], was significantly reduced after heat stress in our study, suggesting that water was directed toward controlling leaf temperature. Even with elevated gs and *E*, the direct exposure to very high temperatures without acclimation resulted in a decrease in the *P*_n_ in coffee EOL, although not in the CL.

Our finding of decreases in net photosynthetic rates in coffee seedlings may be due to damage to a number of cellular functions. Previous studies have established that RuBisCO, the main enzyme for carbon assimilation in plants, exhibits limited activity at high leaf temperatures and undergoes substantial deactivation at very high temperatures [56,57]. In addition, higher temperatures can result in increases in mitochondrial respiration [58]. Thus, the decrease in the non-acclimated *P_n_* in coffee leaves may also be attributed to both RuBisCO impairments and an increase in mitochondrial respiration following heat stress. Acclimation to high temperatures enhances the thermostability of RuBisCO and other associated molecules, leading to effective carbon uptake and improving tolerance to elevated temperatures [57,59]. The maintenance of elevated *P*_n_ values after heat stress in the EOL of acclimated coffee seedlings, as well as the slightly lower C_i_ observed in both AS and REC in these leaves, may be due to the enhanced thermal stability and carboxylation activity of RuBisCO.

At moderately high temperatures, photosynthesis can be significantly reduced, approaching zero, primarily due to RuBisCO deactivation. While some studies have clearly shown that heat can damage the efficiency of PSII [38,39], others have shown that photochemistry is more robust to heat stress than RuBisCO [60]. Under natural weather fluctuations, T_50_ can exceed 54 °C [45,46]. In contrast, seedlings grown under controlled conditions can have a T_50_ ranging between 49 and 50 °C, as observed in here in our control plants and as reported by Marias et al. [44]. Here, we also found that even without acclimation, exposure to stressful temperatures increased the T_15_ and T_50_ of PSII for both CL and EOL after heat stress. Thus, we can conclude that PSII exhibits remarkable PHT adjustments in coffee leaves according to growth conditions or by ontogeny stage.

This study indicates that, based on the parameters examined, the photosynthetic apparatus of 105-day-old coffee seedlings recovered five days after heat stress. In contrast, Marias et al. (2017a) [41] showed that *C. arabica* seedlings older than 6–8 months required at least 15 days to restore gas exchange and Fv/Fm to the levels of non-stressed seedlings. This suggests that younger plants are better able to prevent heat damage or can more quickly repair any heat-induced cellular damage. It has been proposed that young seedlings have a heightened dependency on immediate carbon uptake and fewer storage reserves compared to adult plants, and that adult plants have a lower degree of dependence on immediate carbon uptake after stress [61]. Consistent with this hypothesis, we observed that early EOL that experienced a significant decrease in *P*_n_ AS could recover *P*_n_ to the levels of non-stressed seedlings at REC. It has also been shown in *C. arabica* that leaf discs from mature leaves exhibit higher PHT than those from young leaves [44,46]. All these results indicate that the response to heat stress and the recovery period in *C. arabica* depend on the ontogenetic stage and that additional studies on the stress responses across all developmental stages would be beneficial.

The results presented here suggest that seedling organismal heat tolerance may rely on the photosynthetic responses of cotyledons to heat stress. Here, we report that *C. arabica* cotyledons possess higher photosynthetic heat tolerance compared to EOL, and thus corroborate the trend of the higher photosynthetic activity and tolerance of cotyledons found in other studies [62,63,64]. It has long been established that photosynthetic cotyledons provide carbohydrates essential to the leaf and root development at early growth stages [63,65,66]. More recently, studies in both *Ricinus* (Euphorbiaceae) and *Suaeda* (Chenopodaceae) have demonstrated that during abiotic stress, cotyledons can significantly alter their gene expression and produce higher levels of molecules protective of photosynthesis compared to other types of leaves [64,67]. Here, we demonstrate the maintenance of similar *P*_n_ between heat-stressed and control CL leaves. This suggests that photosynthetic metabolism in CLs is robust to heat stress and may play an important role in organismal-level thermotolerance. Previous studies have shown that transcriptional induction of heat tolerance factors in the shoot apical meristem of *A. thaliana* is based on cotyledons providing not only metabolites (e.g., sucrose) but also systemic signals for meristem functionality under heat stress [68]. Our data is consistent with the hypothesis that the EOLs benefit from the presence of CL leaves and their higher photosynthetic activity under high temperature stress.

## 5. Conclusions

The present study provides insights into the response of *Coffea arabica* to heat stress at the early developmental stage through the evaluation of cotyledons and true leaves from the orthotropic axis. The results show that acclimation to elevated temperatures plays a crucial role in maintaining photosynthetic performance after heat stress. Despite the positive effect of acclimation, differences in the response of *P*_n_ to heat stress compared to PHT were observed, with PSII showing more resilience to heat stress than carbon fixation reactions. We demonstrate here that acclimation preserved the photosynthetic activity and promoted a more efficient recovery of the photosynthetic apparatus after exposure to extreme heat. Importantly, we found that cotyledons generally exhibited higher heat tolerance than true leaves. This distinction underscores the role of cotyledons in facing stressful conditions during the initial development of coffee seedlings.

## Figures and Tables

**Table 1:** Contrasting cotyledons and early-emitted orthotropic leaves’ gas exchange, chlorophyll fluorescence, and photosystem II heat tolerance after stress (AS) and five days after heat stress (REC) by T-test (*p* < 0.05).

Trait	Treatment	After Stress (AS)			Five Days After Stress (REC)		
		Cotyledons	Orthotropic Leaves	*p*	Cotyledons	Orthotropic Leaves	*p*
*P*_n_ ^1^	Control (25 °C)	1.21 ± 0.29	1.39 ± 0.08	0.6261	1.52 ± 0.42	1.57 ± 0.27	0.8379
	48 °C (2 h)	0.95 ± 0.08 **a**	−0.60 ± 0.02 **b**	0.0023	1.03 ± 0.25	0.65 ± 0.19	0.0758
	48 °C (4 h)	0.76 ± 0.26 **a**	−0.38 ± 0.17 **b**	0.0140	0.98 ± 0.19	0.70 ± 0.48	0.6545
	38 + 48 °C (1 h + 2 h)	0.99 ± 0.24	0.71 ± 0.21	0.3985	1.76 ± 0.22	2.17 ± 0.15	0.1258
	38 + 48 °C (1 h + 4 h)	2.36 ± 0.54 **a**	1.22 ± 0.22 **b**	0.0401	1.32 ± 0.27	1.81 ± 0.38	0.1424
*g*_s_ ^2^	Control (25 °C)	14.4 ± 2.16 **b**	22.2 ± 0.83 **a**	0.0264	18.3 ± 3.26	21.3 ± 4.71	0.1884
	48 °C (2 h)	115 ± 7.18	120 ± 16.7	0.7973	23.4 ± 8.67	28.1 ± 14.4	0.5409
	48 °C (4 h)	156 ± 7.65	141 ± 7.40	0.3502	24.8 ± 8.36	19.0 ± 9.27	0.7398
	38 + 48 °C (1 h + 2 h)	154 ± 14.8	159 ± 13.2	0.7028	27.0 ± 5.34 **b**	48.6 ± 3.42 **a**	0.0149
	38 + 48 °C (1 h + 4 h)	110 ± 12.6	122 ± 2.55	0.4574	18.2 ± 5.35	26.2 ± 7.08	0.2187
*E* ^3^	Control (25 °C)	0.19 ± 0.03 **b**	0.30 ± 0.01 **a**	0.0254	0.25 ± 0.04	0.31 ± 0.05	0.1078
	48 °C (2 h)	1.52 ± 0.09	1.59 ± 0.22	0.7816	0.31 ± 0.11	0.38 ± 0.19	0.4876
	48 °C (4 h)	2.05 ± 0.09	1.85 ± 0.09	0.3385	0.33 ± 0.11	0.25 ± 0.12	0.7378
	38 + 48 °C (1 h + 2 h)	2.01 ± 0.18	2.07 ± 0.16	0.7095	0.36 ± 0.07 **b**	0.64 ± 0.04 **a**	0.0159
	38 + 48 °C (1 h + 4 h)	1.45 ± 0.16	1.60 ± 0.03	0.4472	0.24 ± 0.07	0.35 ± 0.09	0.2246
C_i_^4^	Control (25 °C)	272 ± 8.03	290 ± 6.87	0.2935	264 ± 14.6	267 ± 8.83	0.8890
	48 °C (2 h)	384 ± 5.81 **b**	401 ± 5.56 **a**	0.0002	295 ± 22.8	317 ± 33.0	0.5124
	48 °C (4 h)	383 ± 2.68	387 ± 8.67	0.6432	306 ± 25.9	333 ± 18.4	0.2206
	38 + 48 °C (1 h + 2 h)	379 ± 3.57	383 ± 2.92	0.4406	275 ± 19.0	318 ± 3.67	0.1465
	38 + 48 °C (1 h + 4 h)	357 ± 4.52 **b**	374 ± 3.30 **a**	0.0011	266 ± 13.5	278 ± 4.39	0.4205
WUE ^5^	Control (25 °C)	6.32 ± 1.05	4.76 ± 0.32	0.3052	5.97 ± 0.68	5.26 ± 0.32	0.4835
	48 °C (2 h)	0.50 ± 0.19 **a**	−0.48 ± 0.26 **b**	0.0013	4.49 ± 1.07	3.17 ± 1.28	0.3252
	48 °C (4 h)	0.36 ± 0.12 **a**	−0.22 ± 0.10 **b**	0.0148	4.01 ± 1.21	2.72 ± 0.86	0.2375
	38 + 48 °C (1 h + 2 h)	0.53 ± 0.16	0.37 ± 0.13	0.4415	5.42 ± 0.88	3.41 ± 0.17	0.1443
	38 + 48 °C (1 h + 4 h)	1.52 ± 0.19 **a**	0.76 ± 0.15 **b**	0.0006	5.70 ± 0.53	5.30 ± 0.22	0.5070

Bold letters: statistical difference by T-test. Mean ± SEM. ^1^. Pn units: (μmol (CO_2_)m^−2 s−1^); ^2^. gs units: (mol H_2_O m^−2 s−1^); ^3^. E units: (mmol H_2_O m^−2 s−1^); ^4^. Ci units: (μmol CO_2_ mol^−1^ air); ^5^. WUE units: (μmol CO_2_ mmol H_2_O^−1^).

**Table 2:** Contrasting cotyledons and early-emitted orthotropic leaves’ chlorophyll fluorescence and photosystem II heat tolerance (PHT) after stress (AS) and five days after heat stress (REC) by T-test (*p* < 0.05).

Trait	Treatment	After Stress (AS)			Five Days After Stress (REC)		
		Cotyledons	Orthotropic Leaves	*p* Value	Cotyledons	Orthotropic Leaves	*p* Value
Fv/Fm	Control (25 °C)	0.74 ± 0.01 **a**	0.71 ± 0.00 **b**	0.0434	0.74 ± 0.01 **a**	0.71 ± 0.01 **b**	0.0164
	48 °C (2 h)	0.64 ± 0.05 **a**	0.30 ± 0.05 **b**	0.0030	0.69 ± 0.01	0.67 ± 0.04	0.2662
	48 °C (4 h)	0.53 ± 0.06	0.52 ± 0.10	0.9460	0.66 ± 0.01	0.67 ± 0.04	0.6788
	38 + 48 °C (1 h + 2 h)	0.64 ± 0.10	0.64 ± 0.07	0.5973	0.73 ± 0.02 **a**	0.67 ± 0.01 **b**	0.0061
	38 + 48 °C (1 h + 4 h)	0.64 ± 0.03 **a**	0.53 ± 0.08 **b**	0.0361	0.74 ± 0.01 **a**	0.70 ± 0.02 **b**	0.0222
T_15_ (°C)	Control (25 °C)	43.7 ± 0.30 **a**	41.8 ± 0.30 **b**	0.0101	42.5 ± 1.11	42.9 ± 1.32	0.7176
	48 °C (2 h)	*	*	0.6823	46.7 ± 3.26	42.8 ± 2.10	0.3065
	48 °C (4 h)	*	*	0.6252	42.8 ± 1.05	41.3 ± 1.15	0.2805
	38 + 48 °C (1 h + 2 h)	45.7 ± 1.11 **a**	43.0 ± 0.60 **b**	0.0303	46.8 ± 1.10	46.3 ± 1.22	0.4483
	38 + 48 °C (1 h + 4 h)	*	*	0.3484	47.9 ± 0.90	44.6 ± 1.74	0.1343
T_50_ (°C)	Control (25 °C)	49.9 ± 1.10	49.3 ± 0.50	0.2926	50.1 ± 0.38	49.5 ± 0.77	0.3104
	48 °C (2 h)	53.1 ± 0.74	*	0.4472	51.4 ± 0.94 **a**	48.7 ± 0.83 **b**	0.0071
	48 °C (4 h)	50.2 ± 0.51	49.2 ± 4.15	0.6232	51.1 ± 0.46	49.8 ± 1.40	0.1612
	38 + 48 °C (1 h + 2 h)	54.3 ± 1.31 **a**	51.7 ± 0.71 **b**	0.0135	52.6 ± 0.46	52.1 ± 0.90	0.6200
	38 + 48 °C (1 h + 4 h)	51.2 ± 1.24	49.9 ± 3.54	0.1338	54.3 ± 0.52 **a**	51.5 ± 1.29	0.0136

Bold letters: statistical difference by T-test. Mean ± SD. * no values calculated—PSII already damaged at the percentages 15 or 50%.

## Data Availability

All data is available on request.

## Supplementary Materials

The following supporting information can be downloaded at https://www.mdpi.com/article/10.3390/biology14121659/s1; Figure S1: Maximum quantum yield (Fv/Fm) evaluated in PHT assay with Junior-PAM in cotyledonary (blue) and orthotropic (orange) leaves of seedlings subjected to different heat stress treatments. Panel (A) shows Fv/Fm values measured immediately after stress (AS), and panel (B) shows values after a recovery period (REC). Bars represent mean ± standard deviation (SD); Table S1: One-way ANOVA summary for measurements taken after stress (AS); Table S2: One-way ANOVA summary for measurements five days of recovery at the greenhouse condition (REC).

## Abbreviations

AS	After heat stress
REC	5 days of recovery after heat stress
CL	Cotyledons
EOL	Early orthotropic leaves
PSII	Photosystem II
PHT	Photosynthetic heat tolerance
*P_n_*	Net photosynthetic rate µmol CO_2_ m^−2^ s^−1^
*E*	Transpiration rate mmol H_2_O m^−2^ s^−1^
*g_s_*	Stomatal conductance mol H_2_O m^−2^ s^−1^
C_i_	Intercellular CO_2_ concentration µmol mol^−1^
PPFD	Photosynthetic photon flux density µmol photons m^−2^ s^−1^
CO_2_	Atmospheric CO_2_ concentration µmol mol^−1^
WUE	Water use efficiency µmol CO_2_ mmol^−1^ H_2_O
Fv/Fm	Maximum quantum yield of PSII
T_15_	Temperature at which there is a 15% reduction in Fv/Fm compared to initial values
T_50_	Temperature at which there is a 50% reduction in Fv/Fm compared to initial values
