# Supplementary material for: Short-Term Heat Stress Differentially Affects the Photosynthetic Thermotolerance of Cotyledons and Early Orthotropic Leaves in Coffea arabica L. Seedlings
Source: Biology (Basel). 2025 Nov 24;14(12):1659. doi: 10.3390/biology14121659 (PMC12730330; doi:10.3390/biology14121659)
Supplement: Supplementary file 1 [file biology-14-01659-s001.zip › biology-3950640-supplementary.pdf]

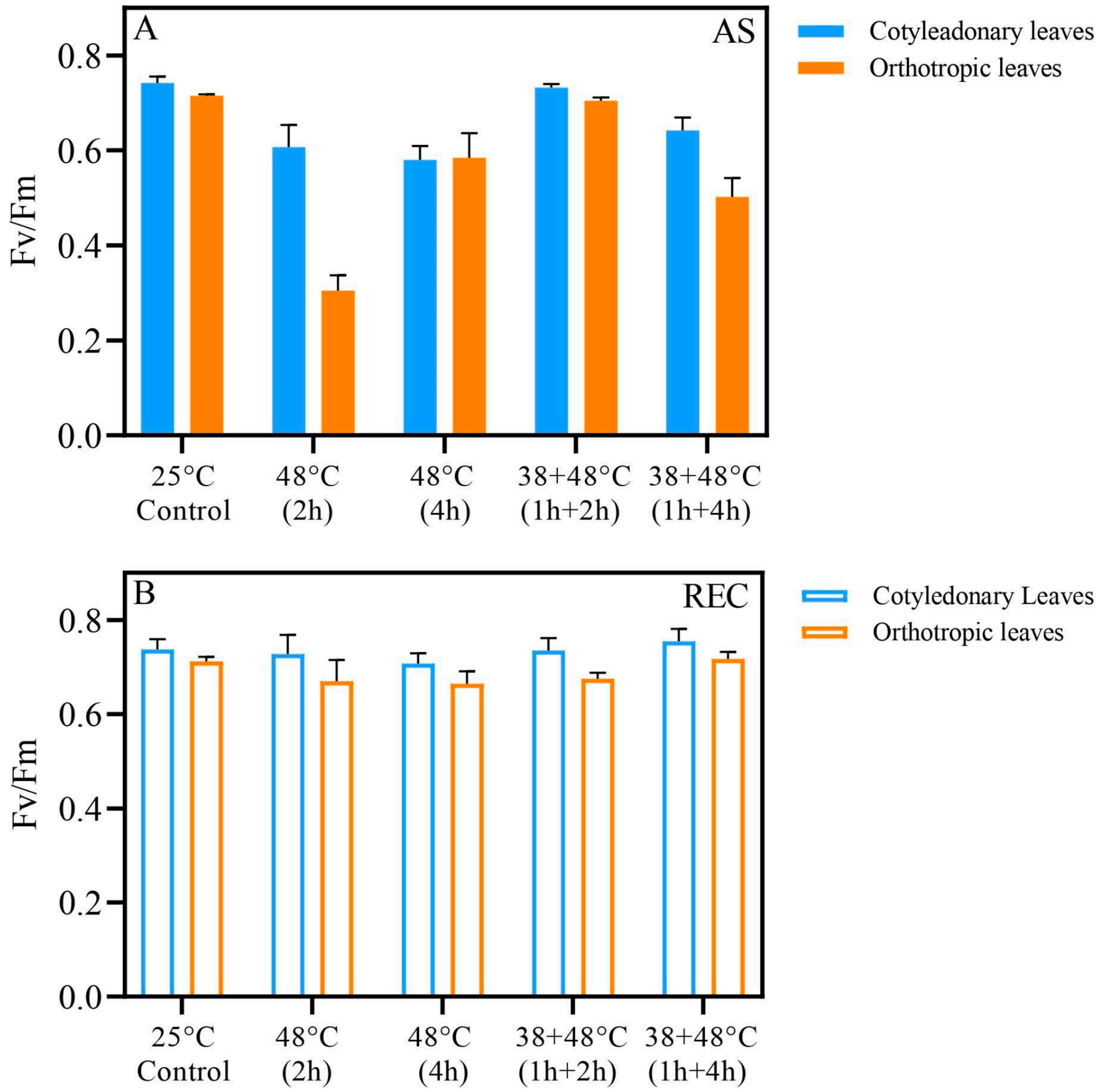

Figure S1. Maximum quantum yield (Fv/Fm) evaluated in PHT assay with Junior-PAM in cotyledonary (blue) and orthotropic (orange) leaves of seedlings subjected to different heat stress treatments. Panel (A) shows Fv/Fm values measured immediately after stress (AS), and panel (B) shows values after a recovery period (REC). Bars represent mean ± standard deviation (SD).

Supplementary Table S1. One-way ANOVA summary for measurements taken after stress (AS). P_n - net photosynthetic rate; g_s - stomatal conductance; E - transpiration; C_i - intercellular carbon WUE - water use efficiency; Fv/Fm - maximum quantum yield; T_{15} and T_{50} - temperature that causes 15% and 50% of decrease in Fv/Fm ratio.

Parameters	Cotyledonary Leaves			Orthotropic Leaves		
	F	p	DFn, DFd	F	p	DFn, DFd
P_n (μmol (CO_2) m^{-2} s^{-1})	3.891	0.0231	4, 15	21.75	<0.0001	4, 15
g_s (mol H_2O m^{-2} s^{-1})	33.64	<0.0001	4, 15	27.59	<0.0001	4, 15
E (mmol H_2O m^{-2} s^{-1})	35.61	<0.0001	4, 15	28.18	<0.0001	4, 15
C_i (μmol CO_2 mol^{-1} air)	81.64	<0.0001	4, 15	56.95	<0.0001	4, 15
WUE (μmol CO_2 mmol H_2O $^{-1}$)	26.40	<0.0001	4, 15	102.1	<0.0001	4, 15
Fv/Fm	6.322	0.0035	4, 15	18.22	<0.0001	4, 15
T_{15} (°C)	5.077	0.0086	4, 15	7.410	0.0017	4, 15
T_{50} (°C)	8.659	0.0008	4, 15	12.27	0.0001	4, 15

Bold numbers: $p<0.05$; DFn: Degree of freedom between the columns; DFd: Degree of freedom within the columns

Supplementary Table S2. One-way ANOVA summary for measurements five days of recovery at the greenhouse condition (REC). P_n - net photosynthetic rate; g_s - stomatal conductance; E - transpiration; C_i - intercellular carbon WUE - water use efficiency; Fv/Fm - maximum quantum yield; T_{15} and T_{50} - temperature that causes 15% and 50% of decrease in Fv/Fm ratio.

Parameters	Cotyledonary Leaves			Orthotropic Leaves		
	F	p	DFn, DFd	F	p	DFn, DFd
P_n (µmol (CO_2) m^{-2} s^{-1})	1.338	0.3015	4, 15	4.452	0.0143	4, 15
g_s (mol H_2O m^{-2} s^{-1})	0.367	0.8286	4, 15	1.766	0.1883	4, 15
E (mmol H_2O m^{-2} s^{-1})	0.369	0.8271	4, 15	1.701	0.2020	4, 15
C_i (µmol CO_2 mol^{-1} air)	0.875	0.5017	4, 15	2.642	0.0751	4, 15
WUE (µmol CO_2 mmol H_2O^{-1})	0.835	0.5239	4, 15	2.899	0.0582	4, 15
Fv/Fm	3.662	0.0284	4, 15	14.89	<0.0001	4, 15
T_{15} (°C)	12.36	0.0001	4, 15	6.428	0.0032	4, 15
T_{50} (°C)	5.510	0.0062	4, 15	11.40	0.0002	4, 15

Bold number: p<0.05; DFn: Degree of freedom between the columns; DFd: Degree of freedom within the columns
